# Infinite-Dimensional Quantum Entropy: The Unified Entropy Case

**DOI:** 10.3390/e26121070

**Published:** 2024-12-09

**Authors:** Roman Gielerak, Joanna Wiśniewska, Marek Sawerwain

**Affiliations:** 1 Institute of Control & Computation Engineering, University of Zielona Góra, Licealna 9, 65-417 Zielona Góra, Poland; r.gielerak@issi.uz.zgora.pl; 2 Institute of Information Systems, Faculty of Cybernetics, Military University of Technology, Gen. S. Kaliskiego 2, 00-908 Warszaw, Poland; jwisniewska@wat.edu.pl

**Keywords:** quantum entropies, unified entropy, Fredholm determinants, numerical determinants

## Abstract

Infinite-dimensional systems play an important role in the continuous-variable quantum computation model, which can compete with a more standard approach based on qubit and quantum circuit computation models. But, in many cases, the value of entropy unfortunately cannot be easily computed for states originating from an infinite-dimensional Hilbert space. Therefore, in this article, the unified quantum entropy (which extends the standard von Neumann entropy) notion is extended to the case of infinite-dimensional systems by using the Fredholm determinant theory. Some of the known (in the finite-dimensional case) basic properties of the introduced unified entropies were extended to this case study. Certain numerical examples for computing the proposed finite- and infinite-dimensional entropies are outlined as well, which allowed us to calculate the entropy values for infinite Hilbert spaces.

## 1. Introduction

Entropy [1] is one of the most significant tools in information theory, both in the classical and quantum approaches [2]. To simplify a bit, entropy in the quantum context, which is considered in this work, describes the level of randomness applied as a quantitative measure of entanglement [3] in an analysed quantum state, which is broadly used in different fields of quantum computation.

Von Neumann entropy is the most popular notion, so it is also termed quantum entropy. There are several important extensions of the entropy notions, in particular, conditional entropy and relative entropy, which play crucial roles in quantum information theory. In addition, we may also apply modifications of the original von Neumann entropy notions, such as quantum–MIN entropy, Tsallis entropy, Rényi entropy, and unified entropies (which are discussed in this work). It is a basic fact that in the infinite-dimensional case, the introduced quantum entropy notions are, in general, not continuous in the full spaces of quantum states. It is very important that the entropy value may be finite or infinite. If we describe quantum states as finite density matrices and utilise the most (and many others as well) popular computational model—quantum circuits—the value of entropy is finite.

However, a continuous-variable quantum computation model (termed as CVQCM, see [4,5,6]) is also considered a very important and highly usable computational model, where a system’s states are represented in infinite Hilbert spaces. An example where the use of a continuous model to describe quantum states is necessary is the physical implementation of quantum calculations, e.g., using a laser or photonics [7]. Another example where the continuous model is an important alternative to computations performed on qubits are computations performed using Gaussian states [8]. Other examples of CVQCM applications include Josephson metamaterials, the quantum noise problem [9], and the Bose–Einstein condensate [10]. Some selected problems, such as the microwave frequency domain, where a two-mode squeezing effect can be observed [11], or the problem of nuclear spin in quantum dots [12], also relate to the use of the CVQCM. In the mentioned cases, the entropy value might also be infinite.

So, in general, our motivations refer to the following two problems: how to calculate the value of entropy when the quantum state is described by a continuous Hilbert space, and how to determine a finite approximation of the entropy when the used definition of entropy indicates that entropy is infinite. For such cases, we discuss a renormalised variant of unified entropy, which allows for computing finite entropy values for states given by the infinite-dimensional Hilbert space with the use of Fredholm determinants [13] technique.

The approach presented in this work enabled us to calculate the entropy values by the standard linear algebra packages—we show exemplary numerical computations carried out with the use of Python code supported by the libraries NumPy [14] and EntDetector [15], which are dedicated to calculating the level of quantum entanglement. The EntDetector package also contains functions that compute entropy values by the technique of Fredholm determinants, both for finite and infinite cases. The examples of the proposed computational methods, presented later in this article, in some cases also allow for bypassing exponential complexity in, e.g., the entropy calculation for the case of two-mode squeezed Gaussian states, although this requires providing an appropriate kernel function. Additionally, the use of the Fredholm approach provides better numerical stability of the calculations than the known direct formula for the entropy value for a bipartite Gaussian state. This is also an important result and motivation for a more thorough investigation of the Fredholm determinants technique, which is presented in this paper.

This paper is organised as follows: in Section 1.1, we present foundations concerning the utilised notation. The entropy notion for bipartite systems is defined in Section 1.2. The methods for calculating and renormalising von Neumann entropy with the use of Fredholm determinants technique is outlined in Section 2. The notion of unified entropy for the finite and infinite cases is introduced in Section 3. In Section 4, we present some numerical examples. Conclusions are contained in Section 5. The Acknowledgments and References end this paper.

### 1.1. On the Standard Notation Used

Sets of real, complex, and integer numbers are denoted as R, C, and N, respectively. Small letters, such as *d*, *i*, *j*, *k*, *l*, and *n*, always stand for integer numbers and are used to denote indexes, dimensionality, or cardinal/ordinal numbers. By the letter H, we always denote a separable Hilbert space; dim(H)≤∞ stands for its dimension. The $C^{⋆}$-algebra of bounded linear operators acting in H is denoted as B(H), and the operator norm as ||·||. The set of all states on H is marked as E(H) (where the notion of a density matrix is used to describe a given state) and its boundary consisting of pure states is denoted as ∂E(H) (in this case, instead of a density matrix, a state vector can be used to describe a quantum state). The multiplicative group of unitary maps acting in H is denoted as U(H). By $B_{+}\left(H\right)$, we denote the set of bounded and positive operators acting on space H: $B_{+}\left(H\right)=\left\{Q\in B\left(H\right):Q\geq 0\right\}$.

The symbol $\mathrm{Tr}\left(\cdot \right)$ stands for the standard trace map defined in the trace-class compact operators acting in H. The corresponding operator’s ideals equipped with the standard Schatten norm $L_{p}$, p≥1, is denoted as $L_{p}\left(H\right)$. For $Q\in L_{p}\left(H\right)$, the spectrum of *Q* is always denoted as $\sigma \left(Q\right)=\left(\lambda_{n}\right)$, n=1,…,∞, and sorted in such way that $\lambda_{i}\geq \lambda_{i+1}$.

If $H=H^{A}⊗H^{B}$, then the corresponding partial trace taking quantum operations is denoted as ${\mathrm{Tr}}_{A}\left(\cdot \right)$ and ${\mathrm{Tr}}_{B}\left(\cdot \right)$, respectively. In particular, if Q∈E(H), then the corresponding reduced density matrices are denoted as $Q_{B}$ and $Q_{A}$, respectively.

### 1.2. Entropy-Based Entanglement Measures in Bipartite Systems

Let us consider a bipartite system “A+B”, the Hilbert space of states that is given as $H=H^{A}⊗H^{B}$, with $\dim H^{A}\cdot \dim H^{B}\leq \infty$.

A map H:E(H)⟶[0,∞] is called an entropy-like map iff (if and only if)

$e_{1}$:  The map *H* is concave (or convex) and finite (or continuous in $L_{1}\left(H\right)$-norm) on E(H);
$e_{2}$:  $\forall_{Q\in \partial E(H)}H\left(Q\right)=0$;
$e_{3}$:  $\forall_{U\in U(H)}H\left(UQU^{†}\right)=H\left(Q\right)$;
$e_{4}$:  *H* is non-increasing under the action of quantum operations;
$e_{5}$:  If $H=H^{A}⊗H^{B}$, Q∈E(H), then $|H\left(Q_{A}\right)-H\left(Q_{B}\right)|\leq H\left(Q\right)$.

Being motivated mainly by the work [16], a map E

(1) E:E(H)⟶[0,∞],

is called an *H*-entropy-based entanglement measure iff it obeys the following properties:

$E_{1}$:  For Q∈∂E(H):
  (2) $$E\left(Q\right)=\left(H\circ {\mathrm{Tr}}_{B}\right)\left(Q\right)=\left(H\circ {\mathrm{Tr}}_{A}\right)\left(Q\right),$$
$E_{2}$:  If Q∈E(H) is separable, then
  (3) E(Q)=0,
$E_{3}$:  E is non-increasing under local quantum operations;
$E_{4}$:  The measure E should be invariant under the action of local unitary groups.

The basic, common elements building the class of entropies discussed in the present note follow a map $I_{r}$ for r∈(0,1)∪(1,∞) (the case of von Neumann entropy corresponding to the choice r=1 is very briefly discussed below in Section 2; see also [17]):

(4) $$\begin{array}{ccc} I_{r}:E\left(H\right) & \to & (0,\infty ],\\ I_{r}\left(Q\right) & = & {\left|\right|Q^{r}\left|\right|}_{1}=\sum_{\lambda \in \sigma (Q)}\lambda^{r}. \end{array}$$

For r≥1, the map $I_{r}$ is exactly the Schatten class operator norm [18] and it is widely used in several applications of the ideals in the operator algebras; see, e.g., [19]. Assuming dim(H)=∞ and r∈(0,1), the situation with the $I_{r}$ definition is much more complicated; see Appendix A. In fact, the following proposition is valid.

**Proposition** **1.** *Let H be a separable Hilbert space with dim(H)=∞ and let r∈(0,1). Then, the set $I_{r}^{\infty }\left(H\right)=\left\{Q\in E\left(H\right):I_{r}\left(Q\right)=\infty \right\}$ is an $L_{1}$-dense subset of E(Q).*

**Proof.**  Let $Q^{\epsilon }$ be a state in E(H), ϵ>0, with the following spectrum $\sigma \left(Q^{\epsilon }\right)={\left(z_{\epsilon }^{-1}\cdot \frac{1}{k^{1+\epsilon }}\right)}_{k}$, $z_{\epsilon }=\sum_{k=1}^{\infty }\frac{1}{k^{1+\epsilon }}<\infty$. Then, for s∈(0,1) obeying $s\leq \frac{1}{1+\epsilon }$, $I_{s}\left(Q^{\epsilon }\right)=\infty$.

Let us choose Q∈E(H) with a spectrum $\sigma \left(Q\right)={\left(\lambda_{k}\right)}_{k}$. For an arbitrarily small δ>0 and arbitrarily large M>0, there exists a number K(δ,M) such that

(5) $$\frac{1}{z_{\epsilon }}\cdot \sum_{k\geq K(\delta ,M)}\frac{1}{k^{1+\epsilon }}<\delta ,$$

and, for $s<\frac{1}{1+\epsilon }$,

(6) $$\sum_{k\geq K(\delta ,M)}\frac{1}{k^{s(1+\epsilon )}}\geq z_{\epsilon }^{s}\cdot M.$$

Now, we form the following spectral set:

(7) $${\left(\sigma_{\delta ,M}\right)}_{k}=z^{-1}\left\{\begin{array}{c} \frac{1}{z_{\epsilon }}\lambda_{k},\;\mathrm{for}\;k\leq K\left(\delta ,M\right),\\ \frac{1}{k^{1+\epsilon }},\;\mathrm{for}\;k>K\left(\delta ,M\right), \end{array}\right.$$

where $z_{\epsilon }=\sum_{k}{\left(\sigma_{\delta ,M}\right)}_{k}<\infty$ uniformly in δ and *M*.

Let $Q{⊗}_{\delta ,M}Q^{\epsilon }$ be any state with a spectrum equal to $\sigma_{\delta ,M}$. Then,

(8) $${||Q-Q{⊗}_{\delta ,M}Q^{\epsilon }\left|\right|}_{1}<\delta ,$$

and $s<\frac{1}{1+\epsilon }$:

(9) $$I_{s}\left(Q{⊗}_{\delta ,M}Q^{\epsilon }\right)=\infty .$$

   □

It is the main motivation for the present paper to propose how to overcome this severe problem that we meet in the case of infinite-dimensional systems.

For this goal, the theory of regularised Fredholm’s determinants was proposed [17] and briefly outlined in the case of standard von Neumann entropy and some two-parameter deformations known under the name unified entropy from Hu and Ye [20]. In the class of entropies analysed in the present paper, the well-known examples of the one-parameter deformations of the von Neumann entropy, widely known as Tsallis and Rényi entropies, are included.

## 2. Quantum von Neumann Entropy and Fredholm Determinants

In this subsection, we outline some of the recent results that we obtained with the use of the Fredholm determinants theory in [17].

Let H be a separable Hilbert space and let dim(H)=∞. For Q∈E(H), it was proved in [17] that the subset

(10) $$\mathrm{vN}\left(\infty \right)=\{Q\in E\left(H\right):\mathrm{Tr}\left(-Q\log Q\right)=\infty \},$$

is a dense subset (in $L_{1}$-topology) in E(H).

For $Q\in vN{\left(\infty \right)}^{c}$, i.e., in the case $\mathrm{Tr}\left(-Q\log Q\right)<\infty$, which is equivalent to $\left(Q^{-Q}-I\right)\in L_{1}\left(H\right)$, it is possible to prove the following Fredholm determinant:

(11) $$D\left(Q\right)=\det(I_{H}+f\left(Q\right)),$$

where $f\left(Q\right)=Q^{-Q}-I_{H}$ is finite, and moreover,

(12) $$\mathrm{vNH}\left(Q\right)=-\mathrm{Tr}\left(Q\log Q\right)=\log D\left(Q\right).$$

Using the technique developed for the analysis of the Fredholm determinants [18,19], it is possible to show that all the basic facts known from quantum von Neumann entropy (11) in the finite-dimensional case, such as, the certain type of continuity, together with properties $e_{1}$–$e_{5}$, besides many others that do hold, can be extended to the subset $vN{\left(\infty \right)}^{c}$ of E(H), where $\mathrm{vN}{\left(\infty \right)}^{c}=E\left(H\right)\setminus \mathrm{vN}\left(\infty \right)$.

Moreover, the following fact can be proved:

(13) $$\mathrm{if}\;Q\in E\left(H\right)\;\mathrm{then}\;\left(Q^{-Q}-I_{H}\right)\in L_{2}\left(H\right).$$

This enables us to write down the following renormalised version of von Neumann entropy:

(14) $${\mathrm{vNH}}^{ren}\left(Q\right)=\mathrm{Tr}\left(-Q\log Q+(Q^{-Q}-I)\right),$$

which appears to be finite and continuous (in the $L_{2}\left(H\right)$-topology) on the whole set of quantum states E(H). The proof is obtained with the use of the regularised Hilbert–Fredholm determinants techniques; see [17].

**Example** **1.** *Let Q∈E(H) be such that $\sigma \left(Q\right)={\left(\lambda_{n}\right)}_{n}=\frac{1}{z_{\beta }}{\left(\frac{1}{n{\left(\log n\right)}^{\beta }}\right)}_{n}$ for β∈(1,∞), $z_{\beta }=\sum_{n=1}^{\infty }\frac{1}{n{\left(\log n\right)}^{\beta }}<\infty$. It is easy to check that $-\sum \lambda_{n}\log\lambda_{n}=\infty$ for β∈(1,2). However, the renormalised entropy is*

(15) $$H^{\mathrm{ren}}\left(Q\right)=\mathrm{Tr}\left(-Q\log Q+(Q^{-Q}-I)\right)=\log\det\left(I_{H}+\left(Q^{-Q}+I\right)\right)e^{-\mathrm{Tr}\left(Q^{-Q}-I\right)}<\infty .$$

*For more on this, see [17].*

## 3. The Unified Quantum Entropies in Terms of the Fredholm Determinants

In this section, we develop a theory of a quantum unified entropy expressed by the use of Fredholm determinants. We recall the Hu–Ye unified entropy (termed HY entropy) and, in the next subsections, we introduce definitions for infinite-dimensional cases with the Fredholm determinants to rewrite unified entropy to obtain finite-dimensional approximations.

### 3.1. The Hu–Ye Unified Entropy for the Finite-Dimensional Case

Let us recall the notion, together with some basic properties, of the two-parameter deformation of the von Neumann entropy, as given in [20] by Xinhua Hu and Zhongxing Ye. Let d=dim(H)<∞. For r∈(0,1)∪(1,∞) and s∈R∖{0}, the Hu–Ye unified entropy $HY_{r}^{s}$ is defined as

(16) $$HY_{r}^{s}:E\left(H\right)\to \left[0,\infty \right),$$

and

(17) $$HY_{r}^{s}\left(Q\right)=\frac{1}{(1-r)s}({\left(\mathrm{Tr}\left(Q^{r}\right)\right)}^{s}-1).$$

Some of the known basic, albeit selected, properties of this quantum entropy version are collected in the next Section 3.2.

### 3.2. HY Entropy Summary (d<∞)

We recall following basic properties of the HY entropy.

$H_{1}$:  Connection with other entropies:
  (i) (18) $$\lim_{s\to 1}HY_{r}^{s}\left(Q\right)=HY_{r}^{1}\left(Q\right)=T_{r}\left(Q\right),$$
    where $T_{r}$ stands for the Tsallis entropy functional and the limit is taken pointwise on E(H).
  (ii) For any admissible value of *r* and s=1:
    (19) $$\lim_{r\to 1}HY_{r}^{s}\left(Q\right)=H\left(Q\right),$$
  (iii) And for any admissible value of *r*:
    (20) $$\lim_{s\to 0}HY_{r}^{s}\left(Q\right)=R_{r}\left(Q\right),$$
    where $R_{r}\left(Q\right)=\frac{1}{1-r}\log\left(\mathrm{Tr}\left(Q^{r}\right)\right)$ is the Rényi entropy.
$H_{2}$:  Non-negativity and boundness for any admissible values of *r* and *s*:
  (21) $$\forall_{Q\in E(H)}\;\;0\leq HY_{r}^{s}\left(Q\right)\leq \frac{1}{(1-r)s}\left(d^{(1-r)s}-1\right)$$
  and:
  (i) (22) $$HY_{r}^{s}\left(Q\right)=0,\;\;\mathrm{iff}\;\;Q\in \partial E\left(H\right),$$
    if $\mathrm{Tr}\left(Q^{2}\right)=1$ (see [20]),
  (ii) (23) $$HY_{r}^{s}\left(Q\right)\leq \frac{1}{(1-r)s}\left({d^{\prime }}^{(1-r)s}-1\right),$$
    iff $\sigma \left(Q\right)=\left(\frac{1}{d^{\prime }},\ldots ,\frac{1}{d^{\prime }},0,\ldots ,0\right)$, where $d^{\prime }=\mathrm{rank}\left(Q\right)$.
$H_{3}$:  If $H=H_{A}⊗H_{B}$, then for any Q∈E(H):
  (i)
(24) $$\forall_{U\in U(H)}\;\;HY_{r}^{s}\left(U^{†}QU\right)=HY_{r}^{s}\left(Q\right),$$
  (ii) Let
    (25) $$Q_{A}={\mathrm{Tr}}_{B}\left(Q\right),\;\;\;Q_{B}={\mathrm{Tr}}_{A}\left(Q\right),$$
    then,
    (26) $$HY_{r}^{s}\left(Q_{A}\right)=HY_{r}^{s}\left(U_{A}^{†}Q_{A}U_{A}\right)\;\mathrm{and}\;HY_{r}^{s}\left(Q_{B}\right)=HY_{r}^{s}\left(U_{B}^{†}Q_{B}U_{B}\right),$$
    where $U_{A(B)}\in U\left(H_{A(B)}\right)$.
  (iii) if $Q\in \partial E(H_{A}⊗H_{B})$, then for any admissible (r,s):
    (27) $$HY_{r}^{s}\left(Q_{A}\right)=HY_{r}^{s}\left(Q_{B}\right).$$
$H_{4}$:  Continuity. It is known [20] that for r>1 and s≥1:
  (28) $$|HY_{r}^{s}\left(Q\right)-HY_{r}^{s}\left(Q^{\prime }\right)|\leq \frac{1}{r(r-1)}{||Q-Q^{\prime }\left|\right|}_{1}.$$
$H_{5}$:  Concavity. Let r∈(0,1), s>0, and r·s<1 or r≥1, r·s≥1, and let $Q=\sum_{k}\lambda_{k}Q_{k}$, $Q_{k}\in E\left(H\right)$, $\lambda_{k}\in \left[0,1\right]:\sum_{k}\lambda_{k}=1$. Then (see [20]),
  (29) $$HY_{r}^{s}\left(Q\right)\geq \sum_{k}\lambda_{k}HY_{r}^{s}\left(Q_{k}\right).$$
$H_{6}$:  Triangle inequality. Let $H=H_{A}⊗H_{B}$ and $Q\in E(H_{A}⊗H_{B})$. Then, for r>1 and $s\geq r^{-1}$ (see [21]):
  (30) $$|HY_{r}^{s}\left(Q_{A}\right)-HY_{r}^{s}\left(Q_{B}\right)|\;\leq \;HY_{r}^{s}\left(Q\right).$$

### 3.3. The Hu–Ye Entropy in an Infinite-Dimensional Case

It will be assumed in the present subsection that dim(H)=∞ and the Hilbert space H is separable. Let, for r>0,

(31) $$\begin{array}{ccc} f_{r}:E\left(H\right) & ⟶ & B_{+}\left(H\right),\\ Q & ⟶ & e^{Q^{r}}-I. \end{array}$$

**Lemma** **1.** *(1) For any Q∈E(H), r≥1,*

(32) $$1\leq {\left|\right|f_{r}\left(Q\right)\left|\right|}_{1}\leq e.$$

*(2) For r∈(0,1), ${\left|\right|Q^{\alpha }\left|\right|}_{r\alpha }^{r\alpha }\leq {\left|\right|f_{r}\left(Q\right)\left|\right|}_{\alpha }^{\alpha }\leq e^{\alpha }{\left||Q|\right|}_{r\alpha }^{r\alpha }$ for any $\alpha \geq \frac{1}{r}$.*

**Proof.**  From the elementary estimate for λ∈[0,1]:

(33) $$\lambda \leq e^{\lambda }-1=\int_{0}^{1}e^{\tau \lambda }\lambda \;d\tau \leq e\cdot \lambda ,$$

it follows that

(34) $$\sum_{\lambda \in \sigma (Q)}\lambda^{r}\leq \sum_{\lambda \in \sigma (Q)}\left(e^{\lambda^{r}}-1\right)\leq e\cdot \sum_{\lambda \in \sigma (Q)}\lambda^{r}.$$

Assuming r≥1, the inequality Equation (32) follows. In the case r∈(0,1) and for any α such that αr>1:

(35) $$\sum_{\lambda \in \sigma (Q)}\lambda^{r\alpha }\leq {\left|\right|f_{r}\left(Q\right)\left|\right|}_{\alpha }^{\alpha }=\sum_{\lambda \in \sigma (Q)}{\left(e^{\lambda^{r}}-1\right)}^{\alpha }\leq e^{\alpha }\cdot \sum_{\lambda \in \sigma (Q)}\lambda^{\alpha r}.$$

   □

#### 3.3.1. The Case r>1

As a corollary of Lemma 1 and the results of [18,22] and the refs. therein, we have the following proposition:

**Proposition** **2.** *Let Q∈E(H) and r≥1. Then, the Fredholm determinant defined as*

(36) $$\begin{array}{ccc} D_{r}:Q\in E\left(H\right) & ⟶ & \langle 1,\infty ),\\ D_{r}\left(Q\right) & ⟶ & \det(I+f_{r}\left(Q\right)), \end{array}$$

*exists (is finite) and obeys the following properties:*

(i) *For any Q∈E(H),*
(37) $$1\leq D_{r}\left(Q\right)\leq e^{e},$$
(ii) *If ${\left(Q_{n}\right)}_{n=1,\ldots }$ is a sequence of states, i.e., $Q_{n}\in E\left(H\right)$, such that*
(38) $$\lim_{n\to \infty }{\left|\right|f_{r}\left(Q_{n}\right)-f_{r}\left(Q\right)\left|\right|}_{1}=0,$$
  *then*
(39) $$\lim_{n\to \infty }D_{r}\left(Q_{n}\right)=D_{r}\left(Q\right),$$
(iii) *The following equalities are valid:*
(40) $$\begin{array}{ccc} D_{r}\left(Q\right) & = & e^{\mathrm{Tr}\left(\log(I+f_{r}\left(Q\right))\right)\;} \end{array}$$
(41) $$\begin{array}{ccc} & = & \sum_{n\geq 0}{\mathrm{Tr}}_{\Lambda^{n}\left(H\right)}\left(\Lambda^{n}\left(f_{r}\left(Q\right)\right)\right), \end{array}$$
  *where $\Lambda^{n}\left(H\right)$ is the n-th skew tensor power of H.*

**Proof.**  For any $Q\in L_{1}\left(H\right)$, the following estimate is known:

(42) $$1\leq \left|\det\left(I+Q\right)\right|\leq e^{{\left||Q|\right|}_{1}},$$

Equations (40) and (41) play a fundamental role in proving Equation (42) (see [18]).

Let r≥1 and Q∈E(H). Then,

(43) $$1\leq |\det\left(I+f_{r}\left(Q\right)\right)|<e^{e},$$

by Lemma 1.

Now, we prove the $L_{1}$-continuity from (ii). For this goal, let us recall some elementary facts:

($f_{1}$)  $L_{1}\left(H\right)$ is a two-sided ⋆-ideal in the $C^{⋆}$-algebra of bounded linear operators acting in H and equipped with the operator norm ||·||. For any A∈B(H) and $B\in L_{1}\left(H\right)$, the following estimates are valid:
  (44) $$\begin{array}{ccc} {\left||AB|\right|}_{1} & \leq & \left||A|\right|\cdot {\left||B|\right|}_{1}\\ {\left||BA|\right|}_{1} & \leq & \left||A|\right|\cdot {\left||B|\right|}_{1} \end{array}$$
($f_{2}$)  If $Q\in L_{1}\left(H\right)$, then
  (45) $$\left||Q|\right|=\underset{\lambda \in \sigma (Q)}{\sup}\left|\lambda \right|,$$
  (see, e.g., [18]).

Using the Duhamel operator formula:

(46) $$e^{Q^{r}}-e^{Q_{n}^{r}}=\int_{0}^{1}e^{\tau Q^{r}}\left(Q^{r}-Q_{n}^{r}\right)e^{\left(1-\tau \right)Q_{n}^{r}}d\tau$$

and from Equation (44):

(47) $${\left|\right|e^{Q^{r}}-e^{Q_{n}^{r}}\left|\right|}_{1}\leq e^{o(1)}{\left|\right|Q^{r}-Q_{n}^{r}\left|\right|}_{1},$$

which proves (ii). To obtain Equation (47), we additionally use the following fact ($f_{2}$), which is valid for compact operators.    □

**Remark** **1.** *The first formula for $D_{r}\left(Q\right)$ in (iii) is known as Plemelj’s formula [23]. The second one is the famous Grothendieck formula [24] used in [18] as one of the basic tools applied there.*

**Remark** **2.** *Assuming r>1, it is not difficult to show that*

(48) $$\lim_{n\to \infty }{\left|\right|e^{Q^{r}}-e^{Q_{n}^{r}}\left|\right|}_{1}=0\;\;\;\mathrm{iff}\;\;\;\lim_{n\to \infty }{||Q-Q_{n}^{r}\left|\right|}_{1}=0.$$

With the use of the introduced determinants, we can rewrite the unified entropy formula of Hu–Ye. If r>1, s≠0, then

(49) $$\forall_{Q\in E(H)}\;{\mathrm{HY}}_{r}^{s}\left(Q\right)=\frac{1}{(r-1)s}\left({\left(\log\left(D_{r}\left(Q\right)\right)\right)}^{s}-1\right).$$

One of the main results presented here is contained in the theorem below.

**Theorem** **1.** *Let dim(H)=∞ and H be separable. Then, for r > 1 and a suitable value of s≠0, let all the properties listed as $H_{1}$, $H_{2}$ (with the form independent of d), HY(3), HY(4), HY(5), and HY(6) be true for ${\mathrm{HY}}_{r}^{s}$ on E(Q).*

**Proof.**  Let Q∈E(H) and let

(50) $$Q=\sum_{\lambda \in \sigma (Q)}\lambda E_{\lambda },$$

be the spectral decomposition of *Q* ($E_{\lambda }$ stands for the orthogonal projectors onto eigenspaces corresponding to λ). For n∈N, we define a finite-dimensional approximation of order *n* to *Q* as

(51) $$Q_{n}=\sum_{k=1}^{n}\lambda_{k}E_{\lambda_{k}},$$

for a natural numeration of λ∈σ(Q) (see the notation from Section 1.1). Then, using the previous Proposition 2:

(52) $$\lim_{n\to \infty }{\left|\right|Q^{r}-Q_{n}^{r}\left|\right|}_{1}=0,$$

and

(53) $$e^{Q^{r}}-e^{Q_{n}^{r}}=\int_{0}^{1}e^{\tau Q^{r}}\left(Q^{r}-Q_{n}^{r}\right)e^{\left(1-\tau \right)Q_{n}^{r}}d\tau ,$$

we obtain

(54) $$\lim_{n\to \infty }\left|\right|f_{r}\left(Q\right)-f_{r}\left(Q_{n}\right){\left|\right|}_{1}=0.$$

Using the $L_{1}$-continuity of Fredholm determinants (see, i.e., [18]), we obtain

(55) $$\lim_{n\to \infty }{HY}_{r}^{s}\left(Q_{n}\right)=HY_{r}^{s}\left(Q\right),$$

for s≠0 and r>1.

Now, we note that for any n∈N, all the properties $H_{1}$–$H_{3}$, $H_{5}$, and $H_{6}$ from Section 3.2 are valid for any n<∞, s≠0, and r≥1.

The continuity $H_{4}$ from Section 3.2 must to be replaced by

${H^{\prime }}_{4}$:  for any r>1, s≥1, and $Q,Q^{\prime }\in E\left(H\right)$:
  (56) $$|HY_{r}^{s}\left(Q\right)-HY_{r}^{s}\left(Q^{\prime }\right)|\;\leq \;o\left(1\right){||Q-Q^{\prime }\left|\right|}_{1}.$$

   □

**Example** **2.** *Let ζ be the zeta function of Riemann, i.e.,*

(57) $$\zeta \left(q\right)=\sum_{n=1}^{\infty }\frac{1}{n^{q}},\;\mathrm{for}\;q>1.$$

*Let P be the set of primes. The operator $Q_{q,r}^{\zeta }\in E\left(H\right)$ is such that*

(58) $$\sigma \left(Q_{q,r}^{\zeta }\right)={\left(z^{-1}{\left(\log\left(1+\frac{1}{p_{k}^{q}}\right)\right)}^{1/r}\right)}_{k},\;\;\;\mathrm{where}\;\;\;z=\sum_{k=1}^{\infty }{\left(\log(1+\frac{1}{p_{k}^{q}})\right)}^{1/r},$$

*which is finite for s>1 and r>1, and $p_{k}\in P$ is the k-th prime (numbered as k-th in the natural ordering of P). Then, by the use of the following formula:*

(59) $$\prod_{p\in P}\left(1+\frac{1}{p^{q}}\right)=\frac{\zeta (q)}{\zeta (2q)},$$

*it follows that*

(60) $$D_{r}\left(Q_{q,r}^{\zeta }\right)=\frac{\zeta (q)}{\zeta (2q)},$$

*and therefore, for s=1,*

(61) $$\frac{\zeta (q)}{\zeta (2q)}=e^{\left(\left(r-1\right)H_{r}^{1}\left(Q_{q,r}^{\zeta }\right)+1\right)}.$$

*Defining the corresponding $Q_{s,r}^{\zeta }$ Hamiltonian:*

(62) $$h_{q,r}^{\zeta }=\log Q_{q,r}^{\zeta }\geq 0,$$

*which is self-adjoint and positive, we enrich the class of Hamiltonians, the spectrum of which is connected to the zeta function of Riemann [25,26,27].*

#### 3.3.2. The Case r∈(0, 1)

Let $0<\delta <\delta^{\prime }\leq 1$. Then, for any Q∈E(H), ${\left|\right|Q^{\delta^{\prime }}\left|\right|}_{1}\leq {\left|\right|Q^{\delta }\left|\right|}_{1}$ (see Appendix A). In particular, for any δ∈(0,1), ${\left|\right|Q^{\delta }\left|\right|}_{1}\geq 1$. For further use, we define for δ∈(0,1) the following sets $E_{\delta }\left(H\right)=\left\{Q\in E\left(H\right):\mathrm{Tr}\left(Q^{\delta }\right)<\infty \right\}$. Then:

(i) For any δ∈(0,1], $E_{\delta }\left(H\right)\neq \emptyset$;
(ii) If $0<\delta <\delta^{\prime }\leq 1$, then $E_{\delta }\left(H\right)\subset E_{\delta^{\prime }}\left(H\right)$;
(iii) Let $E_{o}\left(H\right)={⋂}_{0<\delta \leq 1}E_{\delta }\left(H\right)$; then, $E_{o}\left(H\right)\neq \emptyset$.

The following two lemmas are used below.

**Lemma** **2.** *Let Q∈E(H). Then, for any r∈(0,1], the following equivalence is true:*

(63) $$\left(e^{Q^{r}}-I\right)\in L_{1}\left(H\right)\Leftrightarrow Q^{r}\in L_{1}\left(H\right).$$

**Proof.**  From Lemma 1, it follows that

(64) $$\mathrm{Tr}\left(Q^{r}\right)\leq {\left|\right|e^{Q^{r}}-I\left|\right|}_{1}\leq e^{\alpha }\mathrm{Tr}\left(Q^{r}\right).$$

   □

Also, the following lemma is used in our further discussion.

**Lemma** **3.** *Let Q∈E(H). Then, for r∈(0,1] and j∈N, the following is true:*

(65) $${\left(e^{Q^{r}}-I\right)}^{j}\in L_{1}\left(H\right)\Leftrightarrow Q^{rj}\in L_{1}\left(H\right).$$

**Proof.**  Using

(66) $${\left(e^{Q^{r}}-I\right)}^{j}=\int_{0}^{1}\ldots \int_{0}^{1}e^{\left(\sum_{\alpha =1}^{j}\tau_{\alpha }\right)Q^{r}}\cdot Q^{rj}\;d\tau_{1}\ldots d\tau_{j},$$

we obtain

(67) $${\left|\right|Q^{rj}\left|\right|}_{1}\leq {\left|\right|{\left(e^{Q^{r}}-I\right)}^{j}\left|\right|}_{1}\leq e^{j||Q^{r}\left|\right|}\cdot {\left|\right|Q^{rj}\left|\right|}_{1}.$$

   □

For Q∈E(H), we define its divergence index ${\mathrm{in}}_{HY}$ as

(68) $${\mathrm{in}}_{HY}\left(Q\right)=\underset{0<\delta \leq 1}{\inf}\left\{\mathrm{Tr}\left(Q^{\delta }\right)<\infty \right\}.$$

Thus, taking any Q∈E(H) and $\delta \left(Q\right)={\mathrm{in}}_{HY}\left(Q\right)$, we obtain the following: for any $\delta^{\prime }>\delta \left(Q\right)$, $\mathrm{Tr}\left(Q^{\delta^{\prime }}\right)<\infty$, and for any $\delta^{\prime }<\delta \left(Q\right)$, $\mathrm{Tr}\left(Q^{\delta^{\prime }}\right)=\infty$. Therefore, the corresponding HY entropies as given by Equation (17) are infinite for all values of $0<r<{\mathrm{in}}_{HY}\left(Q\right)$. This is the point where the use of Fredholm determinants enables us to propose a systematic way to extract the finite part of the HY entropy also for values $r\leq {\mathrm{in}}_{HY}\left(Q\right)$.

Let us take Q∈E(H). Then, for any $\delta <{\mathrm{in}}_{HY}\left(Q\right)$, $\sum_{\lambda \in \sigma (Q)}\lambda^{\delta }=\infty$, and therefore, the corresponding HY entropies (as given by Equation (49)) take values equal to infinity.

Originally, the Fredholm determinants were defined on the Banach spaces $L_{1}\left(H\right)$. However, already in the early stage of the Fredholm determinants theory development (mainly addressed to the so-called Fredholm-type integral equations theory), the necessity of extending this theory to spaces $L_{p}\left(H\right)$ for p>1 had originated. Today, the corresponding extensions to the standard ideals of compact operators acting on a general separable Hilbert spaces are known [19,28].

Let us assume that $Q\in L_{1}\left(H\right)$. Then, the Fredholm determinant of *Q* is given by the following formula:

(69) $$\det\left(I+zQ\right)=e^{\left(\sum_{j=1}^{\infty }\frac{{\left(-1\right)}^{j+1}z\mathrm{Tr}\left(Q^{j}\right)}{j}\right)},$$

and (see Equations (40) and (41)) the series under the exponential function *e* is convergent for sufficiently small |z|. Roughly, in the case $Q\in L_{n}\left(H\right)$ and $Q\notin ({⋃}_{j=1}^{n-1}L_{j}\left(H\right))$, the regularised Fredholm determinant is defined as

(70) $$\det_{n}^{ren}\left(I+zQ\right)=e^{\left(\sum_{j=n}^{\infty }\frac{{\left(-1\right)}^{j+1}z\mathrm{Tr}\left(Q^{j}\right)}{j}\right)}.$$

In particular, if $Q\in L_{2}\left(H\right)$, then

(71) $$\det_{2}^{ren}\left(I+zQ\right)=e^{\left(-z\mathrm{Tr}\left(Q\right)\right)}\det\left(I+zQ\right).$$

**Remark** **3.** *The attempts to renormalise the notion of the Fredholm determinants to cover the case of bigger classes of operators have been performed since the beginning of this theory. The extension of the Fredholm theory to the case of the $L_{2}$ class of kernels was published in 1904 by David Hilbert [29]. It was the first paper that introduced the concept of the regularisation of the Fredholm determinants. This idea was further developed in [30], and presently, it is a standard tool of the abstract theory of Hilbert–Schmidt class operators. Generalisations corresponding to the extension of the Fredholm determinants to the higher-order Schatten class are well described in [28].*

Let Q∈E(H), with the divergence index ${\mathrm{in}}_{HY}\left(Q\right)=r_{⋆}$. Then, we define for any $0<r\leq r_{⋆}$:

(72) $$n_{⋆}\left(Q,r\right)=\inf\left\{n\in N:nr>r_{⋆}\right\}.$$

From Lemma 3, it follows that $\mathrm{Tr}\left(Q^{n_{⋆}r}\right)<\infty$, which is equivalent to

(73) $$\mathrm{Tr}\left({\left(e^{Q^{r}}-I\right)}^{n_{⋆}}\right)<\infty .$$

This allows us to define the renormalised Fredholm determinant of $e^{Q^{r}}-I$ by the following formula:

(74) $$\log\left(\det_{n_{⋆}}^{\left(ren\right)}\left(zQ\right)\right)=\sum_{j=n_{⋆}}^{\infty }\frac{{\left(-1\right)}^{j+1}}{j}z^{j}\mathrm{Tr}\left({\left(e^{Q^{r}}-I\right)}^{j}\right).$$

Summarising our discussion, we conclude with the theorem below.

**Theorem** **2.** *Let Q∈E(H) with $0<{\mathrm{in}}_{HY}\left(Q\right)=r_{⋆}<1$.*

(1) *For any $r_{⋆}<r\leq 1$, the following Fredholm determinant:*
(75) $$\det(I+\left(e^{Q^{r}}-I\right))<\infty ,$$
*is well defined on the space $E_{r}\left(H\right)$, and $L_{1}$ is continuous on this space.*
(2) *For any $r\leq r_{⋆}$ and $n_{⋆}$, as defined in Equation (72), the renormalised Fredholm determinant*
(76) $$\det_{n_{⋆}}^{\left(ren\right)}\left(Q\right)=e^{\left(\sum_{j=n_{⋆}}^{\infty }\frac{{\left(-1\right)}^{j+1}}{j}\mathrm{Tr}\left({\left(e^{Q^{r}}-I\right)}^{j}\right)\right)}$$
*is finite and obeys the bound*
(77) $$|\det_{n_{⋆}}^{\left(ren\right)}\left(Q\right)|\;\leq \;e^{o\left(n_{⋆}\right){\left||(e^{Q^{r}}-I)|\right|}_{n_{⋆}}^{nr_{⋆}}}.$$

**Proof.**  According to Lemma 3: ${\left(e^{Q^{r}}-I\right)}^{j}\in L_{1}\left(H\right)$ for any $j\geq n_{⋆}\left(Q\right)$. Therefore, all terms appearing in Equation (76) are finite. The convergence of the series appearing in Equation (76) and the estimate Equation (77) follows straighforwardly from Theorem 6.4 in [18].    □

Let us define now the following operator (for a given Q∈E(H) and r∈(0,1)) for n∈N:

(78) $$R_{n}\left(Q,r\right)=\left(e^{Q^{r}}e^{\sum_{j=1}^{n-1}\frac{{\left(-1\right)}^{j+1}}{j}{\left(e^{Q^{r}}-I\right)}^{j}}\right)-I.$$

**Lemma** **4.** *If $\left(e^{Q^{r}}-I\right)\in L_{n}\left(H\right)$, then*

(79) $$\det_{n}^{ren}\left(Q\right)=\det\left(I+R_{n}\left(Q,r\right)\right).$$

**Proof.**  With the use of Lemma 6.1 from [18] and Lemma 3, we know that $R_{n}\left(Q,r\right)\in L_{1}\left(H\right)$ for any $n_{⋆}$ such that $n_{⋆}\cdot r>{\mathrm{in}}_{HY}\left(Q\right)$. Applying Theorem 6.2 in [18], we conclude the proof.    □

**Remark** **4.** *If $r>{\mathrm{in}}_{HY}\left(Q\right)$, then*

(80) $$\det\left(I+f_{r}\left(Q\right)\right)=\det_{n}^{\left(ren\right)}\left(Q\right)\cdot e^{\sum_{k=1}^{n-1}\frac{{\left(-1\right)}^{k}}{k}\mathrm{Tr}\left({\left(f_{r}\left(Q\right)\right)}^{k}\right)}.$$

**Proof.**  Apply formula 6.4 from [18].    □

**Remark** **5.** *A very interesting question is as follows: which of the basic properties of HY entropy known in the finite-dimensional case (see Section 3.2) and the infinite dimensional case for r>1 also survive the here-proposed renormalisation procedure? This hot problem is still under our active investigations.*

## 4. Numerical Examples

In this section, we present three numerical examples related to quantum states for which we calculated the entropy by the use of the Fredholm determinant technique. A source code in the Python language for all the discussed below examples is publicly available in the earlier-mentioned EntDetector package [15]. In the first example, we calculated the von Neumann entropy using Equations (12) and (14). The second example is dedicated to the quantum state X and unified renormalised entropy like in Equations (49) and (79), where for a given dimensionality, we checked the correctness of the triangle inequality ($H_{6}$) with the entropy expressed by Equation (49). In other words, we calculated partial traces for subsystems A and B and compared the subsystems’ entropies with the entropy of the whole quantum state X. In the third example, we studied numerical calculations of the entropy value for the two-mode squeezed Gaussian states.

Before the presentation of the examples, let us notice that the entropy’s computational complexity calculated, e.g., by Equation (80), depends on the computational complexity of the exponentiation function of the matrix and the operator $f_{r}\left(\cdot \right)$. Therefore, if *T* stands for a general computational complexity, where *d* marks a dimension of the operator *Q*, we obtain

(81) $$\begin{array}{c} T\left(d\right)=T_{D}\left(d\right)+T_{E}\left(d\right)+(\sum_{k=1}^{n-1}T_{s}\left(1\right)+T_{\mathrm{Tr}}\left(Q\right)+T_{f}\left(Q\right))\\ =O\left(d^{3}\right)+O\left(d^{3}\right)+\left(\sum_{k=1}^{n-1}O\left(1\right)+O\left(d^{2}\right)+O\left(d^{3}\right))\right)\\ =O\left(d^{3}\right)+\left(n-1\right)O\left(d^{3}\right)=O\left(d^{3}\right), \end{array}$$

where $T_{s}$ means the computational time for constant values, e.g., a fraction; $T_{D}$ is the complexity of calculation of determinant $\det_{n}^{ren}$ (its complexity is also $O(d^{3})$); $T_{E}$ represents the calculation of matrix exponentiation; $T_{\mathrm{Tr}}$ determines the time of calculating a trace; and $T_{f}$ is the complexity of the function $f_{r}$. Generally, the matrix functions may be computed with the use of the spectral decomposition with its complexity $O(d^{3})$, which determines the complexity of the renormalised Hu–Ye entropy (Equation (80)). In a further part of this section, we also present an entropy approximation for the Gaussian bipartite state by a procedure according to [22], which is as follows:
def fredholm_det(K, z, a, b, m):    w,x=gauss_legendre_quadrature(a,b,m)    w = np.sqrt(w)    xi,xj = np.meshgrid(x, x, indexing=’ij’)    d = np.linalg.det( np.eye(m) + z * np.outer(w,w) * K(xi,xj) )    return d
This procedure’s complexity may be described as

(82) $$T\left(d\right)=T_{GLQ}\left(m\right)+T_{s}\left(n\right)+T_{det}\left(d\right)=O\left(m^{2}\right)+O\left(d^{2}\right)+O\left(d^{3}\right)=O\left(d^{3}\right),$$

where *d* represents the dimension of the operator created as an argument for np.linalg.det. The complexity of the determinant calculation by np.linalg.det is denoted as $T_{det}$. The most dominant operation here is calculating the determinant ($T_{det}$) because the kernel function *K* is computed linearly (depending on the number of points in a given quadrature). Meanwhile, calculating the quadrature’s coefficients $T_{GLQ}\left(m\right)$ is characterised by the quadratic complexity depending on the parameter *m*. It should be added that in this computational routine, instead of the use of a high-dimensional *Q* operator, we used the kernel function *K*, which reduced the whole computational complexity in such a case.

### 4.1. The *d*-Dimensional Isotropic State

In the first example, we compare the value of the standard von Neumann entropy and renormalised the von Neumann entropy $H^{ren}$ for a *d*-dimensional isotropic state:

(83) $$Q_{d,p}^{I}=\frac{\left(1-p\right)}{d^{2}}I+p|\phi^{+}\rangle \langle \phi^{+}|,$$

where $-\frac{1}{\left(d^{2}-1\right)}\leq p\leq 1$, and $|\phi^{+}\rangle =\frac{1}{\sqrt{d}}\sum_{j}|j\rangle ⊗|j\rangle$, i.e., an isotropic state, is defined as a mixture of the maximally mixed state and the maximally entangled state.

**Figure 1:**
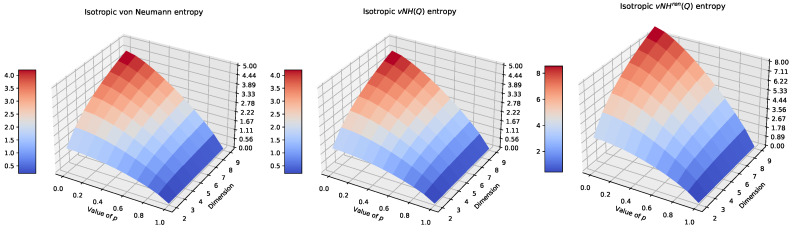
Entropy values (vN, vNH, $vNH^{ren}$) of the isotropic state for selected values of d=2, 3, 4, 5, 6, 7, 8, 9 and p∈[0, 1).

Figure 1 shows the results of entropy computations. In each case, we see the proper asymptotic behaviour of the entropy values for the isotropic case, but naturally, the value of the von Neumann entropy decreased, but in both of the other cases, the values of the renormalised entropies increased.

### 4.2. The *d*-Dimensional X Quantum State

We also examined one of possible generalisations of the X-type quantum state, which can be depicted in a two-form depending on whether the *n* is even $Q_{d}^{Xe}$ or odd $Q_{d}^{Xo}$:

(84) $$Q_{d}^{Xo}=\left[\begin{array}{ccccccccccccc} a_{1} & & & & & & & & & & & & w_{1}\\ \; & a_{2} & & & & & & & & & & ... & \\ \; & & a_{3} & & & & & & & & w_{l} & & \\ \; & & & ... & & & & & & z_{1} & & & \\ \; & & & & ... & & & & ... & & & & \\ \; & & & & & a_{2l} & & z_{l} & & & & & \\ \; & & & & & & a_{2l+1} & & & & & & \\ \; & & & & & z_{l}^{⋆} & & a_{2l+2} & & & & & \\ \; & & & & ... & & & & ... & & & & \\ \; & & & z_{1}^{⋆} & & & & & & ... & & & \\ \; & & w_{l}^{⋆} & & & & & & & & a_{n-2} & & \\ \; & ... & & & & & & & & & & a_{n-1} & \\ w_{1}^{⋆} & & & & & & & & & & & & a_{n} \end{array}\right],$$

(85) $$Q_{d}^{Xe}=\left[\begin{array}{cccccccccccc} a_{1} & & & & & & & & & & & w_{1}\\ \; & a_{2} & & & & & & & & & ... & \\ \; & & a_{3} & & & & & & & w_{l} & & \\ \; & & & ... & & & & & z_{1} & & & \\ \; & & & & ... & & & ... & & & & \\ \; & & & & & a_{2l} & z_{l} & & & & & \\ \; & & & & & z_{l}^{⋆} & a_{2l+1} & & & & & \\ \; & & & & ... & & & ... & & & & \\ \; & & & z_{1}^{⋆} & & & & & ... & & & \\ \; & & w_{l}^{⋆} & & & & & & & a_{n-2} & & \\ \; & ... & & & & & & & & & a_{n-1} & \\ w_{1}^{⋆} & & & & & & & & & & & a_{n} \end{array}\right]$$

where empty cells represent zeros; $n=d^{2}$; and for $a_{i}$, $z_{i}$, and $w_{i}$, we give additional assumptions:

(86) $$\sum_{i=1}^{n}a_{i}=1,\;a_{i}\geq 0,\;\;\;\prod_{i=1}^{l}|z_{i}|\leq \sqrt{\prod_{j=l+1}^{n-l}a_{j}},\;\prod_{i=1}^{l}\left|w_{i}\right|\leq \sqrt{\left(\prod_{j=1}^{l}a_{j}\right)\left(\prod_{j=n-l+1}^{n}a_{j}\right)}.$$

where $l=\left\lfloor \frac{d^{2}}{4}\right\rfloor$.

**Figure 2:**
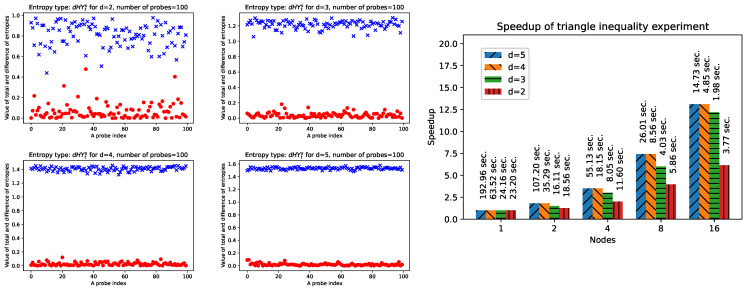
Results of the numerical experiment in which the triangle inequality for unified entropy is verified on state *X*. The charts represent values for one hundred exemplary states *X* with, respectively, dimensions d=2, 3, 4, and 5 (400 samples). Crosses (blue) indicate the entropy of the whole system *X*, while dots mark (red) the absolute values for subsystems A and B of the state *X*. The utilised entropy was $HY_{r}^{s}$ (s = 0.5, r = 2). The chart on the right shows the acceleration gained when the experiment was carried out under the WSL environment for Windows 11 with an AMD Ryzen 9 7950X processor.

Figure 2 depicts the numerical experiment for the state *X* with dimensions 2, 3, 4, and 5, where we check the triangle inequality (HY6) given by Equation (30). This experiment may also be carried out in a parallel environment because each state is checked independently, so it might be realised in the same time. It should be noticed that the experiment’s implementation, thanks to the Python’s environment and auxiliary library EntDetector, needs only a few lines of code, e.g.,m = create_x_state( d )pt0 = ed.PT(m, [d,d], 1 )pt1 = ed.PT(m, [d,d], 0 )ent_m = HY_by_d( m, s, r )ent_pt0 = HY_by_d( pt0, s, r )ent_pt1 = HY_by_d( pt1, s, r )
In the first line, state *X* is calculated with *d* as the dimension of subsystems A and B. In the next line, partial traces are computed (with ed.PT( ) function). Finally, the entropy is obtained according to Equation (49).

### 4.3. The Infinite Bipartite Case

The third example that we would like to present involved calculating the entropy value for the two-mode squeezed Gaussian states given as

(87) $$|\psi_{r}\rangle =\frac{1}{\cosh(r)}\sum_{N=0}^{\infty }\tanh^{N}{\left(r\right)|N\rangle }_{A}⊗{|N\rangle }_{B},$$

where r>0 is the squeezing parameter and |N〉 stands for the “N-particles state”. It is stated in [31] that the formula for the entropy for this state is

(88) $$E_{G}(|\psi_{r}\rangle )=\cosh^{2}\left(r\right)\log\left(\cosh^{2}\left(r\right)\right)-\sinh^{2}\left(r\right)\log\left(\sinh^{2}\left(r\right)\right).$$

**Figure 3:**
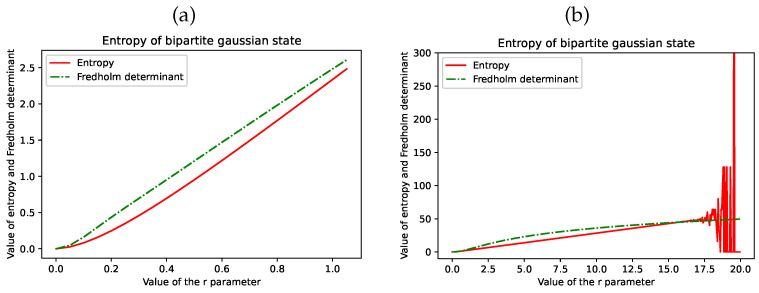
Entropy values for a Gaussian bipartite state (the solid line was calculated according to the analytical equation and the dash–dot line with the use of the Fredholm determinants). Chart (**a**) shows the entropy values for a small range of r∈(0, 1) and chart (**b**) for r∈(0, 20]. We can observe unstable behaviour of the Equation (88) for r≈20, where an overflow floating-point error appears due to the nature of cosh and sinh functions.

The value of entropy diverges to infinity with an increasing value of *r*; however, even for relatively small values of r>17, the above equation may generate an overflow error for computations carried out on double-precision numbers. However, the technique presented in this works together with the procedure fredholm_det, presented at the beginning of Section 4, to correctly approximate the value of $E_{G}(|\psi_{r}\rangle )$. To perform the calculations, we need the kernel function, which, in this case, was

(89) $$K\left(x_{i},x_{j}\right)=\frac{\tanh(x_{i}+x_{j})}{\cosh(x_{i}-x_{j})},$$

where $x_{i}$ and $x_{j}$ are the arguments of the quadrature utilised in the procedure fredholm_det. The technique based on the Fredholm determinants allows for approximating the entropy values with better numerical stability. The results are depicted in Figure 3, where the values of $E_{G}(|\psi_{r}\rangle )$ are calculated for two exemplary ranges of parameter *r*.

## 5. Conclusions

In this article, we show that the Fredholm determinants theory may be successfully applied to calculate the entropy values for finite and infinite cases. This second case is especially important because for states described by the Hilbert space, the values of von Neumann entropy might be infinite. The renormalisation process presented in this paper allows for calculating the approximate entropy value in many cases without any numerical overflow problems.

There are still open issues, e.g., as in Remark 5, which of the basic properties of the renormalised entropy remain true when the parameter r∈(0,1). Further works should also focus on numerical procedures (e.g., the utilisation of other quadratures to better estimate the entropy values for finite and infinite cases).

## Data Availability

Data are contained within the article.

## Appendix A

Several basic properties of p-Schatten operator ideals for p>1 of the $C^{⋆}$-algebras B(H) are described well in many textbooks on Hilbert spaces theory [19,28,32]. However, the fact that most of these properties are also true for p<1 is less known, although various remarks on this topic are dispersed in the literature. The main reason for writing this appendix is to collect some of these properties, which may be useful in the context of the research presented in this article.

Let H be a separable Hilbert space and let $L_{\infty }\left(H\right)$ be the class of all compact operators acting in H with the corresponding norm:

(A1) $${\left||Q|\right|}_{\infty }=\underset{\sigma \in sv(Q)}{\sup}\left\{\sigma \right\},$$

where $sv\left(Q\right)=\left\{\sigma_{1},\sigma_{2},\ldots ,\sigma_{j}\right\}$ denotes the set of singular values of *Q*.

**Definition** **A1.**
*Let p∈(0,∞). A compact operator Σ:H→H is a p-Schatten class operator iff*

(A2)
$$\sum_{j=1}^{\infty }\sigma_{j}{\left(\Sigma \right)}^{p}<\infty .$$

*Then, we define a “p-norm” of Σ:*

(A3)
$${\left||\Sigma |\right|}_{p}^{p}=\sum_{j=1}^{\infty }{\left(\sigma_{j}\left(\Sigma \right)\right)}^{p}.$$

*The set of those $\Sigma \in L_{\infty }\left(H\right)$ such that ${\left||\Sigma |\right|}_{p}<\infty$ are called a p-Schatten class of operators on H and denoted as $L_{p}\left(H\right)$.*

**Remark** **A1.**
*For p∈(0,1), the map $Q\to {\left||Q|\right|}_{p}$ is only a pseudo-norm, as the triangle inequality for ${\left||-|\right|}_{p}$ does not hold (see point (i) in the next Theorem A1).*

**Theorem** **A1.**
*Let p∈(0,1) and H be a separable Hilbert space:*

(i) *If $Q,\Sigma \in L_{p}\left(H\right)$, then $Q+\Sigma \in L_{p}\left(H\right)$ and*
  (A4)
  $$||Q+{\Sigma ||}_{p}^{p}\leq {2||Q||}_{p}^{p}+2{\left||\Sigma |\right|}_{p}^{p}.$$
(ii) *If $Q\in L_{p}\left(H\right)$, then Σ∈B(H),*
  (A5)
  $${\left||Q\Sigma |\right|}_{p}\leq {\left||Q|\right|}_{p}\cdot \left||\Sigma |\right|,$$
  *and*
  (A6)
  $${\left||\Sigma Q|\right|}_{p}\leq \left|\right|Q_{p}\left|\right|\cdot \left||\Sigma |\right|.$$
(iii) *Let $p_{1},p_{2}\in \left(0,1\right)$, $Q\in L_{p_{1}}\left(H\right)$, and $\Sigma \in L_{p_{2}}\left(H\right)$. Then, $Q\Sigma \in L_{p}\left(H\right)$, where $\frac{1}{p}=\frac{1}{p_{1}}+\frac{1}{p_{2}}$ and*
  (A7)
  $${\left||Q|\right|}_{p}\leq 2^{\frac{1}{p}}{\left||Q|\right|}_{p_{1}}\cdot {\left||\Sigma |\right|}_{p_{2}}.$$
(iv) *For 0 < p < q < 1:*
  (A8)
  $$L_{p}\left(H\right)\subset L_{q}\left(H\right).$$
